# Recurrent Immune-Mediated Thrombotic Thrombocytopenic Purpura in the Setting of Suspected Sjögren’s Syndrome

**DOI:** 10.7759/cureus.117022

**Published:** 2026-09-27

**Authors:** Biruk Amare, Sajid Ehsan, Imran Yousaf, Deepak Gupta, Hannah Rupert

**Affiliations:** 1 Division of Rheumatology, Department of Medicine, TidalHealth, Salisbury, USA

**Keywords:** adamts13, anti-ssa, autoimmune disease, immune-mediated thrombotic thrombocytopenic purpura, mycophenolate mofetil, sjögren syndrome, thrombotic thrombocytopenic purpura

## Abstract

A 70-year-old African American female with a past history of relapsing/remitting thrombotic thrombocytopenic purpura (TTP), hypertensive retinopathy, and breast cancer was evaluated in the rheumatology clinic for a possible autoimmune rheumatic disease. Earlier, she had followed up with a community rheumatologist who diagnosed her with systemic lupus erythematosus (SLE) and Sjögren syndrome (SS). She was being treated with hydroxychloroquine, but this was discontinued after her hospitalization for a TTP flare. Investigations revealed the presence of anti-nuclear antibodies (ANA) and anti-SSA antibodies. Anti-double-stranded DNA (dsDNA) and anti-Smith antibodies were not present. Her main complaints were dry eyes and dry mouth, and there were no rashes, mouth sores, alopecia, serositis or polyarthritis. Although she had a positive ANA, the available clinical and serologic documentation did not establish sufficient additional criteria to fulfill the 2019 European Alliance of Associations for Rheumatology/American College of Rheumatology (EULAR/ACR) classification criteria for SLE. Her relapsing TTP was treated with multiple courses of rituximab and plasmapheresis with ADAMTS13 level monitoring. Mycophenolate mofetil (MMF) was added as a steroid-sparing immunosuppressive agent in the setting of recurrent immune-mediated TTP, ongoing corticosteroid exposure, and concomitant autoimmune features suggestive of Sjögren disease. Thereafter, the patient remained stable without another documented TTP relapse during follow-up. This temporal association does not establish that MMF prevented TTP relapse. This case report emphasizes an interesting association between suspected Sjögren disease and recurrent TTP and highlights the diagnostic and therapeutic challenges of managing recurrent TTP in the setting of systemic autoimmune features.

## Introduction

Sjögren syndrome (SS) is a systemic autoimmune disease characterized by sicca symptoms and variable extraglandular manifestations. The 2016 American College of Rheumatology/European Alliance of Associations for Rheumatology (ACR/EULAR) classification criteria use weighted objective findings, with anti-SSA/Ro positivity and a qualifying minor salivary gland biopsy each contributing three points; ocular staining, Schirmer testing, and unstimulated salivary flow each contribute one point [1]. Treatment is guided by the organ manifestations and may include topical therapies, hydroxychloroquine (HCQ) for selected musculoskeletal manifestations, conventional immunosuppressive agents, and biologic therapy for selected systemic disease [2].

Thrombotic thrombocytopenic purpura (TTP) is a life-threatening thrombotic microangiopathy caused by severe ADAMTS13 deficiency, usually mediated by autoantibodies that inhibit the enzyme or increase its clearance, resulting in accumulation of ultralarge von Willebrand factor multimers and platelet-rich microvascular thrombi [3,4]. Acute TTP is treated with therapeutic plasma exchange and corticosteroids, with rituximab and caplacizumab incorporated according to contemporary guidance [5,6]. The coexistence of Sjögren disease and TTP has been described, but the association remains uncommon. A 2021 case-based review identified 18 patients reported through 2020 [7], and a subsequent French registry cohort identified 30 patients with immune-mediated TTP and Sjögren disease, including 28 cases among 1,231 registry patients (2.2%) [8,9].

The coexistence of suspected Sjögren disease and recurrent TTP presents a clinical challenge because sicca symptoms and positive serology may raise suspicion for systemic autoimmune disease even when objective classification criteria are incomplete. In addition, the extent to which Sjögren disease influences the severity, relapse risk, or outcomes of immune-mediated TTP remains uncertain. We present a patient with recurrent objectively confirmed TTP, persistent sicca symptoms, and strong anti-SSA/Ro52/Ro60 seropositivity in whom SS remained clinically suspected because available objective classification criteria were incomplete. This case highlights the uncommon coexistence of TTP and SS and adds to the limited literature describing this association. It also underscores the diagnostic and therapeutic challenges of managing recurrent TTP in the setting of underlying autoimmune features, while recognizing that a causal relationship between SS and TTP cannot be established from this case.

## Case presentation

A 70-year-old woman with a past medical history of right breast invasive ductal carcinoma status post right partial mastectomy, hypertension, hyperlipidemia, reported systemic lupus erythematosus (SLE), and SS presented to the rheumatology clinic after a diagnosis and treatment of TTP. Her first documented episode of TTP was approximately one year before her current presentation, when she presented with severe thrombocytopenia, acute kidney injury (AKI), and worsening anemia. Her ADAMTS13 activity at the time was less than 5%. She was initiated on daily therapeutic plasma exchange (TPE) and high-dose methylprednisolone. She subsequently had persistent TTP despite therapeutic plasma exchange. She was then started on rituximab 375 mg/m² weekly for four doses. This was followed by vincristine 1 mg IV. She ultimately completed 33 plasma exchanges before they were discontinued. Prednisone was gradually tapered as ADAMTS13 recovered to 93%. Unfortunately, three months after completion of plasma exchange and prednisone, she had her first TTP relapse. She required additional plasma exchange at a large academic center. She also received an additional course of rituximab 375 mg/m². Prednisone was restarted. During this time, her TTP resolved and, at the same time, she was evaluated by rheumatology and labeled as having SLE and SS and was started on HCQ 200 mg daily. Laboratory review from that time demonstrated a positive anti-nuclear antibodies (ANA) and positive anti-SSA antibodies, although no clear documentation of additional clinical or serologic criteria establishing SLE was available. Unfortunately, between January and approximately three years after her initial episode, the patient was once again readmitted to the hospital for another recurrent TTP relapse. She was treated with TTP-directed therapy and remained on HCQ. After her hospital discharge, from 2024 to mid-2025, her TTP was quiescent without an additional relapse. She continued to follow with hematology/oncology and outside rheumatology. In the latter half of 2025 (Table 1), the patient had a major TTP relapse where her platelet count declined markedly from approximately 200 × 10³/µL two months earlier to 26 × 10³/µL, with laboratory evidence of microangiopathic hemolysis. The individual laboratory values are provided in Table 1. Her PLASMIC score, a tool used to predict severe ADAMTS13 deficiency in suspected TTP, was 6 [6]. This indicated a high probability of TTP. She did not have neurologic deficits at the time of presentation. She was started on methylprednisolone (Solu-Medrol) 1 g IV daily for three days. Therapeutic plasma exchange was also urgently started because of the high PLASMIC score. Over the course of her hospitalization, her platelets increased to 235 × 10³/µL. Once her platelets remained above 150 × 10³/µL for at least two days, plasma exchange was discontinued and she was subsequently transitioned from IV Solu-Medrol to oral prednisone of approximately 1 mg/kg/day. At the time of discharge, her platelet count had stabilized following TPE and corticosteroid therapy, and she was planned for outpatient weekly rituximab with hematology/oncology. She also requested a second rheumatology opinion for management of her reported SS.

**Table 1:** Laboratory findings during thrombotic thrombocytopenic purpura (TTP) relapse and subsequent rheumatologic evaluation ALT: alanine aminotransferase, AST: aspartate aminotransferase, BUN: blood urea nitrogen, LDH: lactate dehydrogenase, MCV: mean corpuscular volume, WBC: white blood cell

Test	Result	Normal/Reference Range	Interpretation
Hemoglobin	10.0 g/dL	12.0-16.0 g/dL	Low; anemia
MCV	75 fL	80-100 fL	Low; microcytosis
WBC	6.6 × 10³/µL	4.0-11.0 × 10³/µL	Normal
Platelets	26 × 10³/µL	150-450 × 10³/µL	Severely low; thrombocytopenia
BUN	25.3 mg/dL	7-20 mg/dL	Mildly elevated
Creatinine	1.33 mg/dL	0.6-1.2 mg/dL	Elevated; renal dysfunction
Total Bilirubin	1.38 mg/dL	0.2-1.2 mg/dL	Mildly elevated
LDH	848 U/L	120-250 U/L	Elevated; consistent with hemolysis/tissue injury
Reticulocyte Count	0.015	0.5-2.5%	Within reference range
Haptoglobin	<10 mg/dL	30-200 mg/dL	Low; consistent with hemolysis
Peripheral Blood Smear	1-2% schistocytes	No schistocytes	Schistocytes present, consistent with microangiopathic hemolysis
Calcium	10.4 mg/dL	8.5-10.5 mg/dL	High-normal/mildly elevated
AST	45 U/L	10-40 U/L	Mildly elevated
ALT	54 U/L	7-56 U/L	Within/near upper reference range
LDH	549 U/L	120-250 U/L	Elevated

During subsequent rheumatologic reassessment, her diagnoses of SLE and SS were reassessed. Her clinical findings at her first rheumatology appointment included chronic xerostomia and xerophthalmia. There was no reported parotid gland swelling, and the history of an SLE diagnosis remained unclear because the chart primarily documented “Lupus - On Plaquenil.” The available records did not provide sufficient clinical or serologic documentation to establish the basis of the previous SLE diagnosis. Her initial laboratory evaluation included a urine microalbumin/creatinine ratio of 94 mg/g (reference <30 mg/g) and urinalysis positive for 1+ blood. No proteinuria was documented. CBC was grossly within normal limits. Repeat peripheral smear showed slight hypochromia with few ovalocytes and teardrop cells. Her ESR and CRP were within normal limits. Her autoimmune panel was remarkable, as shown in Table 2. Because the main laboratory findings relevant to the TTP relapse are presented in Table 1 and the autoimmune serologies in Table 2, individual laboratory values are not repeated here unless clinically necessary. The combination of sicca symptoms, positive ANA, strongly positive anti-SSA, and elevated Ro52 and Ro60 increased suspicion for suspected Sjögren disease. However, there remained insufficient evidence to definitively establish SLE. The patient did not have a documented history of rashes, oral ulcers, alopecia, serositis, inflammatory polyarthritis, or other clearly attributable clinical manifestations of SLE. Although thrombocytopenia and anemia occurred during TTP episodes, these abnormalities were considered in the setting of objectively documented TTP and were not attributed to SLE. Based on the available information, she did not accumulate sufficient additional points to meet the 10-point threshold required by the 2019 EULAR/ACR SLE classification criteria after the ANA entry criterion was met [8].

**Table 2:** Autoimmune serologic findings obtained during rheumatologic evaluation, including reported patient results, reference values, and interpretation ANA: antinuclear antibody, IgG: immunoglobulin G

Test	Result	Normal/Reference Value	Interpretation
ANA Interpretation	Positive	Negative	Positive
Anti-SSA (Ro) Antibodies	418	<20	Positive
Anti-SSB (La) Antibodies	10	<20	Negative
Sm Antibodies	5	<20	Negative
U1RNP Antibodies	4	<20	Negative
Scl-70 Antibodies	4	<20	Negative
Jo-1 Antibodies	1	<20	Negative
Anti-dsDNA Antibodies	10	<20	Negative
Centromere Antibodies	3	<20	Negative
Histone Antibodies	3	<20	Negative
Ro52 Antibody, IgG	107	<20	Elevated
Ro60 Antibody, IgG	568	<20	Elevated

At her follow-up appointment, she was referred for a minor salivary gland/lip biopsy and ophthalmology evaluation for objective assessment of xerophthalmia. Discussion with hematology at this time approved resumption of HCQ. Repeat ADAMTS13 activity improved to 84% while she was receiving prednisone. Given the patient's history of recurrent immune-mediated TTP, ongoing corticosteroid exposure, and concomitant autoimmune features suggestive of Sjögren disease, mycophenolate mofetil (MMF) 1 g daily was initiated as a steroid-sparing immunosuppressive agent, with a plan to titrate to 1.5 g/day and then 2 g/day. MMF was not considered TTP-directed therapy. Results of her minor salivary gland biopsy showed salivary gland tissue with atrophy and focal mild nonspecific chronic inflammation. The pathologist noted that the findings may occur in SS and required correlation with clinical history and serology.

As a result, the working diagnosis remained suspected Sjögren disease based on sicca symptoms and strong anti-SSA/Ro52/Ro60 positivity. At her most recent follow-up, five months after initiation of MMF, her TTP remained in remission. The patient reported that TTP relapses had become much less frequent after treatment with HCQ and MMF. This patient-reported observation is not interpreted as evidence of causality. Her immunomodulatory treatment included HCQ 400 mg/day and MMF 2 g/day. Her MMF was subsequently reduced to 1.5 g/day due to diarrhea. She was also initiated on pilocarpine for xerostomia. Her overall status remained clinically controlled with no further TTP relapse. The absence of a subsequent documented TTP relapse after MMF initiation represents a temporal association and does not establish that MMF prevented TTP relapse.

## Discussion

SS is a chronic autoimmune disorder of the exocrine glands associated with lymphocytic infiltrates of the affected glands [1]. The hallmark clinical manifestation includes dryness of the mouth and eyes due to dysfunction of the affected glands [1]. Fatigue and pain are also common symptoms. Systemic features, also known as extraglandular manifestations, include arthralgias/arthritis, Raynaud's phenomenon, cutaneous vasculitis, peripheral neuropathy, interstitial lung disease, interstitial nephritis, and immune thrombocytopenia [1,3]. Serologic findings may include anti-Ro/SSA and anti-La/SSB antibodies, positive rheumatoid factor, hypergammaglobulinemia, and hypocomplementemia [1]. The 2016 ACR/EULAR criteria assign a weighted score in patients with signs and symptoms suggestive of the disease [2]. A score greater than or equal to 4 classifies primary SS (Table 3). Based on these criteria, the patient had positive anti-SSA antibodies, which gives her 3 points [2]. Though she had dry eyes and dry mouth, they do not themselves contribute to the classification score [2]. Unfortunately, Schirmer testing and ocular staining score have not yet been performed. Her minor salivary gland biopsy was inconclusive. Therefore, she was labeled as having “suspected Sjögren syndrome.” The original histopathology images from the minor salivary gland biopsy were not available; therefore, the biopsy findings are presented based on the pathology report.

**Table 3:** 2016 ACR/EULAR Classification Criteria for Primary Sjögren Syndrome (SS). ACR/EULAR: American College of Rheumatology/European Alliance of Associations for Rheumatology [2]

Item	Finding	Score
Anti-SSA/Ro antibody positive	Positive serum anti-SSA/Ro antibodies	3
Minor salivary gland biopsy	Focal lymphocytic sialadenitis with a focus score ≥1 focus/4 mm²	3
Ocular staining score	Ocular staining score ≥5 (or van Bijsterveld score ≥4) in at least one eye	1
Schirmer test	≤5 mm/5 minutes in at least one eye	1
Unstimulated whole salivary flow	≤0.1 mL/min	1

Treatment of SS depends on whether its manifestations are non-visceral or visceral. Non-visceral manifestations such as arthralgia and myalgia may be treated with non-steroidal agents and hydroxychloroquine. Systemic therapies may be considered for active systemic disease, and corticosteroids, immunosuppressive agents, and biologic therapies may be selected according to the organ involved and severity [3]. In this patient, MMF was initiated as steroid-sparing immunosuppression in the setting of recurrent immune-mediated TTP, ongoing corticosteroid exposure, and suspected Sjögren disease. MMF was not initiated as TTP-directed therapy. Although MMF has been used in systemic Sjögren disease, evidence supporting its efficacy is limited, and current EULAR recommendations do not identify a preferred immunosuppressive agent. The subsequent absence of TTP relapses after MMF initiation represents a temporal association rather than evidence of a causal effect of MMF. The patient's HCQ dose was later increased to 400 mg/day.

The patient therefore had a total available classification score of 3 points and did not meet the ≥4-point threshold for classification under the 2016 ACR/EULAR criteria. The clinical diagnosis remained suspected Sjögren disease because of her persistent sicca symptoms and strong anti-SSA/Ro52/Ro60 seropositivity despite incomplete objective classification evidence.

On the other hand, TTP is a rare, life-threatening thrombotic microangiopathy. It occurs due to severe deficiency of ADAMTS13, the von Willebrand factor-cleaving protease. ADAMTS13 is a plasma metalloprotease enzyme that regulates hemostasis by cleaving ultra-large von Willebrand factor (UL-vWF) multimers into smaller, less thrombogenic forms [4]. In immune-mediated TTP, autoantibodies against ADAMTS13 lead to severe enzyme deficiency and formation of platelet-rich microthrombi, resulting in thrombocytopenia and microangiopathic hemolytic anemia [4]. The diagnosis of TTP includes obtaining a CBC, examination of peripheral blood smear, reticulocyte count, direct antiglobulin (Coombs) test, lactate dehydrogenase (LDH), prothrombin time (PT), partial thromboplastin time (PTT), fibrinogen, haptoglobin and serum bilirubin (direct and indirect), ADAMTS13 activity, and autoantibody (inhibitor) assays. Just as important is the exclusion of other thrombocytopenic disorders. The PLASMIC score can also provide a pretest probability of severe ADAMTS13 deficiency and help guide treatment decisions [6]. Early recognition is important in patients suspected of having TTP so treatment can be promptly initiated. Although the classic pentad of thrombocytopenia, microangiopathic hemolytic anemia, neurologic abnormalities, renal dysfunction, and fever is historically associated with TTP, the complete pentad is not required for diagnosis and should therefore not delay treatment [7]. In contrast to her uncertain diagnosis of SS, the diagnosis of recurrent TTP in our patient was supported by multiple objective findings. During her initial episode in 2020, she presented with severe thrombocytopenia, acute kidney injury, worsening anemia, and an ADAMTS13 activity level of <5%. She subsequently experienced multiple relapses requiring therapeutic plasma exchange, corticosteroids, rituximab, and, during her initial episode, vincristine. During her major relapse in September 2025, she again demonstrated findings consistent with thrombotic microangiopathy, including marked thrombocytopenia, anemia, biochemical evidence of hemolysis, and schistocytes on peripheral blood smear (Table 1). Her PLASMIC score was 6, indicating a high probability of severe ADAMTS13 deficiency [6]. Subsequent testing demonstrated an ADAMTS13 activity level of <5% with a detectable ADAMTS13 inhibitor, further supporting recurrent acquired immune-mediated TTP. The mainstay treatment of immune-mediated TTP includes therapeutic plasma exchange and corticosteroids, with rituximab and caplacizumab used according to the clinical setting [7,10]. The patient in this case received therapeutic plasma exchange, corticosteroids, rituximab, and other TTP-directed therapy during her relapses.

It is important to note the relationship between Sjogren disease and immune-mediated TTP has been described in the literature but it also remains uncommon. A 2024 French registry cohort identified 30 patients with immune-mediated TTP and Sjogren disease. It is noted that, in comparison with patients with idiopathic immune-mediated TTP, investigators did not identify clear differences in mortality or clinical relapse [8,9]. The overall short-term outcomes were favorable. Interestingly, although patients with Sjogren's disease more frequently require salvage therapy, the data do not establish this, as Sjogren's disease is associated with more severe or relapsing TTP. With this in mind the relatively small cohort and observational data of the study limit the definitive conclusions regarding disease severity and outcome. This case also illustrates the importance of distinguishing an established diagnosis of immune-mediated TTP from a suspected underlying autoimmune rheumatic disease. The patient's current TTP was supported by objective evidence of severe ADAMTS13 deficiency and ADAMTS13 inhibitor, whereas the diagnosis of Sjogren's disease remains suspected because the available objective classification criteria were incomplete. Likewise the prior diagnosis of SLE could not be substantiated from the available records. In summary the subsequent absence of documented TTP relapse after initiation of MMF is clinically interesting but should be interpreted cautiously because the patient also received TTP-directed therapy such as corticosteroids and other immunomodulatory treatment.

## Conclusions

This case describes a patient with recurrent TTP and suspected SS. Although her Sjögren diagnosis could not be definitively confirmed, her persistent sicca symptoms and strong anti-SSA/Ro52/Ro60 positivity raised significant suspicion for the disease. Her TTP remained in remission after treatment with hematology/oncology-directed therapy along with HCQ and MMF. While the absence of further documented TTP relapses was temporally associated with initiation of MMF, this case does not establish that MMF or treatment of suspected Sjögren disease prevented TTP relapse. The available 2024 French registry data likewise do not establish that Sjögren disease is associated with more severe or more relapsing immune-mediated TTP. This case highlights a rare association between these two conditions and the importance of evaluating for underlying autoimmune disease in patients with recurrent TTP while maintaining appropriate caution in attributing changes in disease activity to a specific immunomodulatory therapy.
